# Changes in microvascular perfusion of heart and skeletal muscle in sheep around the time of birth

**DOI:** 10.1113/EP090809

**Published:** 2022-11-24

**Authors:** Matthew W. Hagen, Samantha Louey, Sarah M. Alaniz, Todd Belcik, Matthew M. Muller, Laura Brown, Jonathan R. Lindner, Sonnet S. Jonker

**Affiliations:** ^1^ Center for Developmental Health Oregon Health & Science University Portland OR USA; ^2^ Knight Cardiovascular Institute Oregon Health & Science University Portland OR USA; ^3^ Department of Pediatrics University of Colorado Anschutz Medical Campus Aurora CO USA; ^4^ Division of Cardiovascular Medicine University of Virginia Medical Center Charlottesville VA USA

**Keywords:** development, ultrasound, vascular

## Abstract

Microvascular perfusion of striated muscle is an important determinant of health throughout life. Birth is a transition with profound effects on the growth and function of striated muscle, but the regulation of microvascular perfusion around this transition is poorly understood. We used contrast‐enhanced ultrasound perfusion imaging (CEUS) to study the perfusion of left ventricular myocardium and hindlimb biceps femoris, which are populations of muscle with different degrees of change in pre‐ to postnatal workloads and different capacities for postnatal proliferative growth. We studied separate groups of lambs in late gestation (135 days’ gestational age; 92% of term) and shortly after birth (5 days’ postnatal age). We used CEUS to quantify baseline perfusion, perfusion during hyperaemia induced by adenosine infusion (myocardium) or electrically stimulated unloaded exercise (skeletal muscle), flow reserve and oxygen delivery. We found heart‐to‐body weight ratio was greater in neonates than fetuses. Microvascular volume and overall perfusion were lower in neonates than fetuses in both muscle groups at baseline and with hyperaemia. Flux rate differed with muscle group, with myocardial flux being faster in neonates than fetuses, but skeletal muscle flux being slower. Oxygen delivery to skeletal muscle at baseline was lower in neonates than fetuses, but was not significantly different in myocardium. Flow reserve was not different between ages. Given the significant somatic growth, and the transition from hyperplastic to hypertrophic myocyte growth occurring in the perinatal period, we postulate that the primary driver of lower neonatal striated muscle perfusion is faster growth of myofibres than their associated capillary networks.

## Abstract (highlights)

- **What is the central question of this study?**
  How does the microvascular perfusion of striated muscle change during the dynamic developmental period between the late gestation fetus and early neonate?
- **What is the main finding and its importance?**
  In both myocardium and skeletal muscle, perfusion of striated muscle is significantly reduced in the neonate compared to the late term fetus, but flow reserve is unchanged. The results suggest striated muscle capillary networks grow more slowly relative to the myofibres they nourish during the perinatal period.

## 1 INTRODUCTION

Birth is a major physiological transition affecting the work of striated muscles and consequently their growth. Heart and skeletal muscles are both striated and have similarities and differences with regards to growth patterns and physiological function. Perfusion of the microvasculature in these muscles is essential for proper function at rest and during exercise. How microvascular perfusion and growth are regulated around the time of birth is unknown.

Heart and postural muscles have different balances of pre‐ versus post‐natal demand, with myocardial contraction persisting before and after birth, and hindlimb skeletal muscle engagement occurring primarily after birth. Prenatal perfusion of the myocardium is necessary for both growth and cardiac function, while in skeletal muscle perfusion is entirely uncoupled from work. At birth, cardiac workload shifts rapidly: the loss of the placenta increases systemic vascular resistance, the initiation of air breathing increases arterial oxygen content and decreases pulmonary vascular resistance, and the closure of fetal shunts redistributes systolic load on the ventricles (Morton & Brodsky, 2016). Meanwhile demand on skeletal muscle increases rapidly, with the need for postural control in humans, and the immediate ambulation in species with more precocial young, including sheep.

In addition, both striated muscle populations experience important shifts from proliferative to hypertrophic growth dynamics near birth, with myofibre number for life largely determined prenatally (Austin et al., 1995; Brown, 2014; Burrell et al., 2003; Jonker et al., 2015). Postnatal myocyte proliferation occurs in skeletal muscle through the engagement and fusion of satellite cells, but cardiac myocyte number is determined prenatally, with minimal regenerative capacity even in the context of injury (White et al., 2018; Yin et al., 2013).

Despite the well‐defined courses of myocyte growth during the perinatal period, the development and function of the vascular systems that nourish these growing muscles are less clearly defined on this timescale. In the myocardium, postnatal angiogenesis occurs, but the capacity for adaptive angiogenesis to maintain flow reserve in response to challenges such as left ventricular hypertrophy declines with age (Flanagan et al., 1994, 1991). In contrast, skeletal muscle angiogenesis can occur throughout a healthy lifetime in response to exercise or altitude (Latroche, Gitiaux et al., 2015; Malek et al., 2010; Olfert & Birot, 2011).

We studied the perfusion of two populations of striated muscle: the left ventricular (LV) free wall myocardium and the biceps femoris (BF) hindlimb skeletal muscle in late gestation fetal and neonatal lambs. We used contrast‐enhanced ultrasound perfusion imaging (CEUS) to dynamically measure perfusion kinetics. We have previously successfully used CEUS in a fetal ovine setting (Jonker et al., 2016). CEUS has advantages over techniques such as transit time flow probes and radioactive microspheres including the ability to independently resolve microvascular volume and flux rate, limit invasiveness, and increase anatomical specificity (Kaufmann et al., 2007; Lindner, 2021). We performed CEUS to test our hypothesis that the increased growth, changes in resistance and metabolic demands of postnatal life will lead to an increase in microvascular flow and flow capacity in both muscle groups in the neonate compared to the fetus, with greater differences in microvascular capacity in skeletal muscle than cardiac because of the greater increase in demand.

## 2 METHODS

### 2.1 Ethical approval

All animal experiments were approved by the Institutional Animal Care and Use Committee of Oregon Health & Science University, protocol no. IP00000007, which is accredited by AAALAC International.

### 2.2 Groups and surgical instrumentation

**FIGURE 1:**
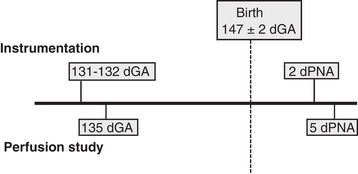
Study timeline. Fetuses were instrumented at 131 or 132 days’ gestational age (dGA) and studied at 135 dGA. Lambs were born at 147 ± 2 dGA, instrumented at 2 days’ postnatal age (dPNA) and studied at 5 dPNA

Time‐bred pregnant ewes of mixed western breed carrying twins were obtained from local vendors. The experimental timeline is illustrated in Figure 1. Common elements of fetal and neonatal surgeries are similar to our earlier work (Jonker et al., 2016).

#### 2.2.1 Sample sizes and exclusions

Five pairs of twin fetuses were instrumented for study. One fetus died from complications related to the surgery in the 24 h following the surgery, resulting in the early termination of the twin pair. Four sets of twin fetuses survived to imaging study. All data from one fetus were excluded because of severe acidaemia during the study, leaving seven fetuses (three male, four female) in our dataset. Adenosine hyperaemia and flow reserve data were unavailable for one fetus because of a non‐functional left atrial infusion catheter, flow reserve data were unavailable for another fetus because of a non‐functional aortic occluder, and electrically stimulated exercise hyperaemia skeletal muscle perfusion and skeletal muscle flow reserve data were unavailable for another because of incorrectly applied electrodes. Therefore, final fetal sample sizes were seven for baseline myocardial and skeletal muscle perfusion studies, six for hyperaemic myocardium, hyperaemic skeletal muscle, and skeletal muscle flow reserve studies, and five for myocardial flow reserve.

Eight lambs from twin pregnancies were instrumented for study, and all survived to the study endpoint. One lamb was excluded due to severe hypoxaemia during the study, leaving seven lambs (three male, four female) in our dataset. Within these, all skeletal muscle data were excluded from one lamb because of poor probe position during the study. Therefore, final neonatal sample sizes were seven for all myocardial studies and six for all skeletal muscle studies.

#### 2.2.2 Fetal instrumentation

Both fetuses in twin pregnancies were instrumented at 131 or 132 days’ gestational age (dGA; term is 147 days, see Figure 1). Maternal anaesthesia was induced with ketamine (400 mg, i.v.) and diazepam (10 mg, i.v.). Ewes were intubated, and mechanically ventilated with a mix of oxygen (2 litres min^−1^), nitrous oxide (0.7 litres min^−1^) and isoflurane (1.5–2%). The uterus was exposed via abdominal midline incision and two separate hysterotomies performed in series to access each fetus in its amniotic sac. Per fetus, two saline‐filled vinyl catheters were placed in one carotid artery (0.86 mm inner diameter (ID) × 1.32 mm outer diameter (OD), 1.19 × 1.80 mm ID × OD, tips advanced to the aortic arch). Another two saline‐filled vinyl catheters were placed in one jugular vein (both 1.19 × 1.80 mm ID × OD, tips advanced to the superior vena cava). Each fetal thoracic cavity was accessed by left thoracotomy. A deflated occluder composed of silicone elastomer (10–12 mm, In Vivo Metric, Healdsburg, CA, USA) was placed around the descending postductal aorta. One saline‐filled vinyl catheter (0.86 × 1.32 mm ID × OD with a 1.80 mm OD cuff on the inserted end) was placed in the left atrium via direct puncture. Catheters were anchored to the fetal skin, along with a vinyl amniotic sac catheter anchored to the fetal skin (1.19 × 1.80 mm ID × OD). Free ends were tunnelled subcutaneously and exteriorized to a pouch sutured on the flank of the ewe. Fetal and maternal incisions were closed in layers and separate doses of ciprofloxacin (2 mg) and penicillin G (1 million units) were delivered to each fetus's amniotic sac. Ewes were returned to floor pens after weaning from anaesthesia and when spontaneously breathing, extubated once swallowing, and continuously monitored until standing and eating, at which point buprenorphine (0.3 mg, s.c.) and slow release buprenorphine (0.05 mg kg^−1^, s.c.) were administered. Ewes had ad libitum access to food and water following recovery.

#### 2.2.3 Neonatal instrumentation

Lambs of both sexes from twin pregnancies were born on‐site by spontaneous vaginal delivery; surgeries were performed at 2 days’ postnatal age (dPNA). Lambs were pretreated with carprofen (2 mg kg^−1^ s.c.) and anaesthesia was induced with ketamine (3 mg kg^−1^, i.v.) and diazepam (0.5 mg kg^−1^, i.v.). Lambs were intubated and mechanically ventilated with oxygen (2 litres min^−1^) and isoflurane (1.5–2%). Lambs were instrumented with vascular catheters, an atrial catheter and a postductal aortic occluder as described above for fetuses. Catheters were tunnelled subcutaneously and exteriorized to the back, secured using body netting and protected with a jacket. Incisions were closed in layers, and air was evacuated from the thoracic cavity using a water‐sealed chest tube and manual positive‐pressure ventilation. Lambs were recovered in the operating room until awake. Following extubation but prior to wakefulness, lambs were given corn syrup on their gums and at least 100 ml expressed sheep milk via a gastric tube. Lambs were continuously monitored until ambulatory and feeding from the ewe. They were given a second dose of 2 mg kg^−1^ carprofen (s.c.) 24 h after the first dose.

### 2.3 Perfusion study

Fetuses and lambs were allowed multiple days to recover from instrumentation surgeries prior to terminal imaging studies. Arterial blood gases were measured daily between instrumentation and study, as described below.

#### 2.3.1 Fetal imaging study

Ewes were anaesthetized as described above and mechanically ventilated with oxygen (2 litres min^−1^) and isoflurane (1.5–2%). The maternal abdominal incision was re‐opened and extended into a Mercedes incision to avoid compression of the umbilical cord. Fetuses were imaged one at a time. Fetuses were exteriorized through a hysterotomy and exposed to the level of the diaphragm for myocardial imaging. For skeletal muscle imaging, one hindlimb was exposed to the hip through a second uterine incision.

#### 2.3.2 Neonatal imaging study

Neonatal anaesthesia was induced and maintained as described for instrumentation above.

#### 2.3.3 Arterial blood contents and characteristics

Arterial blood samples were collected via aortic catheters into heparinized syringes and either processed immediately or stored on ice for up to 2 h prior to processing. The partial pressures of oxygen and carbon dioxide, pH, concentration of haemoglobin, and oxygen, glucose and lactate contents were measured using a blood gas analyser (ABL 825 Radiometer America, Cleveland, OH, USA; temperature adjusted to 39°C); haematocrit was measured by capillary centrifugation.

#### 2.3.4 Contrast‐enhanced perfusion imaging

Fetal and neonatal systemic arterial and venous pressures were recorded by connecting one each of the aortic and superior vena cava catheters to pressure transducers (Transpac, Abbott, Abbott Park, IL, USA) which were connected to a bridge amplifier and recorder (Powerlab, ADInstruments Inc., Colorado Springs, CO, USA) and recorded at 40 Hz. Transducer height was adjusted to the level of the heart. Arterial blood samples were collected immediately before and after imaging and processed as described above. Atropine (0.5 mg kg^−1^) and propranolol (1 mg kg^−1^) were given systemically immediately before imaging to control heart rate.

All ultrasound imaging was carried out using a Sequoia 512 ultrasound scanner (Siemens Medical Systems, Malvern, PA, USA). Myocardial imaging to determine microvascular perfusion in the LV free wall myocardium was performed using a phased array transducer at 1.5 MHz (4V1c‐s Acuson) from a subcostal view, while hindlimb skeletal muscle imaging to determine microvascular perfusion in the BF was performed using a linear array transducer with centreline frequency of 7 MHz (15L8‐s Acuson). The non‐linear signal component for microbubbles was detected using multi‐pulse phase‐and amplitude‐modulation at a mechanical index of 0.18 and a dynamic range of 55 dB. Gain settings were optimized and held constant. One vial of microbubble ultrasound contrast agent (DEFINITY, Lantheus Medical Imaging, North Billerica, MA, USA) was diluted in 15 ml saline and infused systemically via the superior vena cava. Blood pool (*I*_B_) imaging of the LV cavity was performed with an infusion rate of 30 or 60 ml h^−1^. Depending on observed contrast signal, myocardial perfusion imaging was performed at 30 or 60 ml h^−1^, and skeletal muscle imaging was performed at 30, 60 or 90 ml h^−1^. Myocardial perfusion imaging was performed at end systole and skeletal muscle imaging was performed with a 4 Hz trigger. Images were acquired for a minimum of 8 s after a high‐power (mechanical index 1.8) five‐frame destructive pulse sequence. Myocardial imaging always preceded BF imaging.

#### 2.3.5 Imaging conditions

Myocardial imaging was conducted at baseline, with hyperaemia induced by infusion of adenosine into the left atrium (147 μg kg^−1^ min^−1^), and with adenosine hyperaemia and transient inflation of the postductal aortic occluder to transiently increase coronary perfusion pressure. Skeletal imaging was conducted at baseline and during unloaded exercise induced by electrically stimulating the BF with an external pacemaker (10 mA, 2 Hz, 5345 Temporary Pulse Generator, Medtronic Inc., Minneapolis, MN, USA) for at least 60 s before and during imaging. All imaging was performed in at least triplicate.

#### 2.3.6 Imaging analysis

Detailed descriptions of CEUS perfusion imaging analysis have been published (Kaufmann et al., 2007). For perfusion imaging analysis, signal from non‐capillary vessels was eliminated by background subtraction of the first post‐destruction end‐systolic frame (myocardium) or a frame obtained 0.5 s after destruction (BF) from all subsequent frames. Time‐intensity data after the destructive pulse sequence were fitted to the equation:

(1) $$y=A(1-e^{-\beta t})$$

where *y* is intensity at time *t*, *A* is the plateau intensity reflecting relative microvascular blood volume, and the rate constant β represents the microvascular flux rate (s^−1^). Absolute microvascular blood volume (MBV) was quantified by scaled comparison of *A* with *I*_B_:

(2) $$\mathrm{MBV}=A/(1.06\times I_{B}\times F)$$

where 1.06 is tissue density (g cm^−1^) and *F* is the scaling factor to correct for variations in the infusion rates. Microvascular blood flow (MBF; ml min^−1^ g^−1^) is the product of MBV and β, multiplied by 60 (s min^−1^).

### 2.4 Killing

At the conclusion of each fetal imaging study, fetal cardiac arrest in diastole was induced by bolus delivery of saturated potassium chloride (i.v.). Following the second twin's imaging study, ewes were killed with an overdose of a commercial barbiturate (i.v., SomnaSol, Covetrus, Dublin, OH, USA) while still anaesthetized. At the conclusion of neonatal imaging studies, cardiac arrest was induced in lambs with an overdose of the same commercial barbiturate (i.v.) while still anaesthetized. Fetuses and lambs were systemically anticoagulated by bolus delivery of heparin (6000 U, i.v.) immediately prior to killing. Hearts were rapidly excised and weighed whole.

### 2.5 Explanation of parameters

Flux rate (β, s^−1^) is the rate constant describing how quickly the microvasculature in a region of interest fills.

Microvascular blood volume (MBV, ml g^−1^) is the microvascular blood content as a proportion of tissue mass.

Microvascular blood flow (MBF, ml min^−1^ g^−1^) is microvascular blood flow as a proportion of tissue mass.

Arterial minus venous pressure (mmHg) is the difference between mean systemic arterial and venous pressures.

Rate pressure product (RPP, mmHg min^−1^) is an estimate of cardiac work. It is the product of arterial minus venous pressure and heart rate.

Conductance (ml min^−1^ mmHg^−1^ g^−1^) is the relationship between MBF and arterial minus venous pressure.

Flow reserve is the degree to which flow can be increased from baseline by maximal hyperaemia. It is expressed as fold difference. Due to the potential effects of adenosine on systemic pressure, hyperaemic MBF in the myocardium was measured with and without transient aortic occlusion so that a two‐point regression of MBF on arterial minus venous pressure could be generated. Myocardial flow reserve is calculated as the fold change between adenosine‐induced hyperaemic MBF fitted to baseline arterial minus venous pressure and baseline MBF. Skeletal muscle flow reserve is calculated as the fold change between flow measured during electrically stimulated exercise and flow measured at baseline.

Oxygen delivery (ml min^−1^ g^−1^) is the oxygen brought to the muscle of interest by arterial blood. It is calculated as the product of ABF and arterial oxygen content.

### 2.6 Statistics

Results are reported as mean (standard deviation (SD)). All statistics were computed using R (v4.2.0, R Foundation for Statistical Computing, Vienna, Austria) (R Core Team, 2022). All hypothesis tests concerned the differences between fetuses and neonates. Data determined by the Bartlett test to be normally distributed were analysed using a two‐sided unpaired Student's *t*‐test; those determined to be non‐normally distributed were computed using the Mann–Whitney *U*‐test. Data were considered statistically significant when *P* < 0.05

## 3 RESULTS

Fetuses were studied at 135 dGA. The gestational age at birth of the neonatal group was 147 ± 2 dGA. Lambs were studied at 5 dPNA. Therefore, on average, 17 developmental days separated the groups.

### 3.1 Morphometry

Lambs were 75% heavier than fetuses (Table 1) and lamb hearts were more than twice the weight of fetal hearts. The heart to body weight ratio was 25% greater in lambs than fetuses.

**TABLE 1:** Sample sizes and weights

	Fetus	Neonate	*P*
***n* (male)**	7 (3)	7 (3)	
**Body (kg)**	3.6 (0.9)	6.3 (1.0)	0.0002
**Heart (g)**	22.7 (4.6)	50.4 (5.6)	<0.0001
**Heart to body (g kg^−1^)**	6.5 (0.7)	8.1 (0.8)	0.0011

Sample sizes, sex distributions and endpoint body and heart weights for fetuses (135 dGA) and neonates (5 dPNA). Data are means (SD). Differences assessed by *t*‐test.

### 3.2 Blood gases and contents

Blood gases and contents from unanaesthetized animals on the day of CEUS study are reported in Table 2. Arterial pH was similar between neonates and fetuses. The partial pressure of oxygen was 4.4‐fold greater, and the partial pressure of carbon dioxide 18% lower in neonates than fetuses. Haemoglobin, haematocrit and lactate were lower in neonates relative to fetuses, while oxygen and glucose content were both higher.

**TABLE 2:** Blood gases and contents

	Fetus	Neonate	*P*
**pH**	7.36 (0.03)	7.32 (0.07)	0.1953
$P_{CO_{2}}$ (mmHg)	50.1 (2.4)	41.2 (5.7)	0.0051
$P_{O_{2}}$ (mmHg)	19.6 (2.8)	85.9 (12.1)	0.0006
**Haemoglobin (g dl^−1^)**	13.0 (2.1)	9.7 (2.0)	0.0108
**Haematocrit (%)**	43.1 (6.7)	26.3 (4.3)	0.0002
**O_2_ content (mg dl^−1^)**	8.4 (1.6)	12.9 (2.8)	0.0045
**Glucose (mmol l^−1^)**	0.7 (0.2)	6.2 (0.4)	<0.0001
**Lactate (mmol l^−1^)**	2.1 (0.4)	1.4 (0.6)	0.0112

Blood gases and contents measured from unanaesthetized, resting fetuses (135 dGA, *n* = 7) and neonates (5 dPNA, *n* = 7) on the day of CEUS study. Data are means (SD). Non‐parametrically distributed data ($P_{O_{2}}$) assessed by Mann–Whitney *U*‐test; all others assessed by *t*‐test. Differences considered statistically significant when *P* < 0.05.

### 3.3 Study haemodynamics

Fetal and neonatal haemodynamics recorded during anaesthetized imaging studies were similar between fetuses and neonates during all study conditions with the exception of heart rate during adenosine hyperaemia and adenosine hyperaemia with transient occluder inflation studies of myocardial perfusion, when heart rate was 15% faster in neonates than fetuses (Table 3).

**TABLE 3:** Study haemodynamics (under anaesthesia)

		Fetus	Neonate	*P*
Arterial minus venous pressure (mmHg)	Baseline (LV)	38.3 (3.4)	37.9 (5.5)	0.8726
	Adenosine (LV)	32.5 (5.5)	30.5 (5.0)	0.5242
	Ado + occluder (LV)	40.2 (6.7)	39.9 (6.6)	0.9390
	Baseline (BF)	41.5 (6.5)	37.5 (7.2)	0.3232
	Exercise (BF)	43.0 (6.7)	35.2 (7.3)	0.0828
Heart rate (min^−1^)	Baseline (LV)	135 (18)	149 (7)	0.7332
	Adenosine (LV)	131 (20)	151 (8)	0.0350
	Ado + occluder (LV)	133 (20)	152 (8)	0.0480
	Baseline (BF)	137 (6)	148 (6)	0.1266
	Exercise (BF)	135 (18)	149 (6)	0.0931
RPP (mmHg min^−1^)	Baseline (LV)	5174 (861)	5647 (981)	0.3570
	Adenosine (LV)	4325 (1250)	4628 (895)	0.6329
	Ado + occluder (LV)	5434 (1598)	6041 (1040)	0.3642
	Baseline (BF)	5636 (884)	5562	0.9018
	Exercise (BF)	5773 (1036)	5263	0.3939

Arterial minus venous pressure, heart rate, and rate pressure product (RPP) recorded from fetuses (135 dGA) and neonates (5 dPNA) during myocardial perfusion imaging of the left ventricular free wall (LV) and skeletal muscle of the hindlimb biceps femoris (BF). Baseline LV (*n* = 7 fetuses, 7 neonates), adenosine hyperaemia LV (*n* = 6 fetuses, 7 neonates), adenosine hyperaemia with transient aortic constriction (Ado + occluder, *n* = 5 fetuses, 7 neonates), baseline BF (*n* = 7 fetuses, 6 neonates) and electrically stimulated exercise hyperaemia BF (*n* = 6 fetuses, 6 neonates). Non‐parametrically distributed data (heart rate in all LV studies and in exercising BF studies, and RPP in exercising BF studies) assessed by Mann–Whitney *U*‐test; all others assessed by *t*‐test. Differences are considered statistically significant when *P* < 0.05.

### 3.4 Microvascular blood volume

**FIGURE 2:**
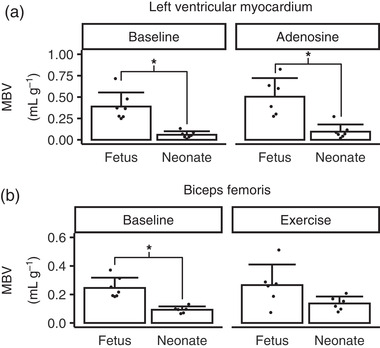
Microvascular volume. Microvascular blood volume (MBV) was assessed in fetuses at 135 dGA and in neonates at 5 dPNA. (a) Microvascular blood volume in the LV free wall myocardium was lower in neonates compared to fetuses at baseline (*n* = 7 fetuses, 7 neonates; Mann–Whitney *U*‐test, *P* = 0.0006) and during adenosine hyperaemia (*n* = 6 fetuses, 7 neonates; Mann–Whitney *U*‐test, *P* = 0.0012). (b) Volume was similarly reduced in the biceps femoris at baseline (*n* = 7 fetuses, 6 neonates; Mann–Whitney *U*‐test, *P* = 0.0012), but was not significantly different during electrically stimulated exercise (*n* = 6 fetuses, 6 neonates; Mann–Whitney *U*‐test, *P* = 0.0931). Column heights correspond to group means, error bars show + SD and dots represent individual values. *P < 0.05.

Microvascular blood volume in the LV free wall myocardium was substantially lower in neonates than fetuses at baseline (Figure 2a, 0.06 (0.04) vs. 0.40 (0.16) ml g^−1^, *P* = 0.0006) and with adenosine hyperaemia (0.10 (0.08) vs. 0.50 (0.22) ml g^−1^, *P* = 0.0012). In BF, microvascular blood volume was likewise significantly reduced in neonates compared to fetuses at baseline (Figure 2b, 0.09 (0.02) vs. 0.25 (0.07) ml g^−1^, *P* = 0.0012). Blood volume was not significantly different between age groups in BF with electrically stimulated exercise hyperaemia (neonates 0.14 (0.05) vs. fetuses 0.27 (0.14) ml g^−1^, *P* = 0.0931).

### 3.5 Microvascular flux rate

**FIGURE 3:**
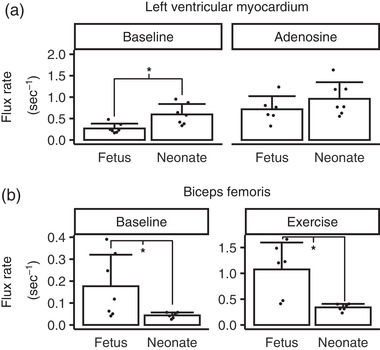
Microvascular flux rate. Microvascular flux rate was assessed in fetuses at 135 dGA and in neonates at 5 dPNA. (a) In the LV myocardium, neonatal flux rate is faster than fetal at baseline (*n* = 7 fetuses, 7 neonates; *t*‐test, *P* = 0.0112) but not different during adenosine hyperaemia (*n* = 6 fetuses, 7 neonates; *t*‐test, *P* = 0.2310). (b) In the BF, neonatal flux rate is significantly slower than fetal at rest (*n* = 7 fetuses, 6 neonates; Mann–Whitney *U*‐test, *P* = 0.0350) and during electrically stimulated exercise (*n* = 6 fetuses, 6 neonates; Mann–Whitney *U*‐test, *P* = 0.0022). Column heights correspond to group means, error bars show + SD and dots represent individual values. *P < 0.05.

Microvascular flux rate in the LV free wall myocardium was significantly greater in neonates compared to fetuses at baseline (Figure 3a, 0.60 (0.24) vs. 0.27 (0.11) s^−1^, *P* = 0.0112), but did not significantly differ with adenosine hyperaemia (0.96 (0.39) vs. 0.72 (0.31) s^−1^, *P* = 0.2310). Conversely, flux rate was lower in neonatal relative to fetal BF at baseline (Figure 3b, 0.04 (0.01) vs. 0.18 (0.14) s^−1^, *P* = 0.0350) and during electrically stimulated exercise (0.34 (0.07) vs. 1.08 (0.52) s^−1^, *P* = 0.0022).

### 3.6 Microvascular blood flow

**FIGURE 4:**
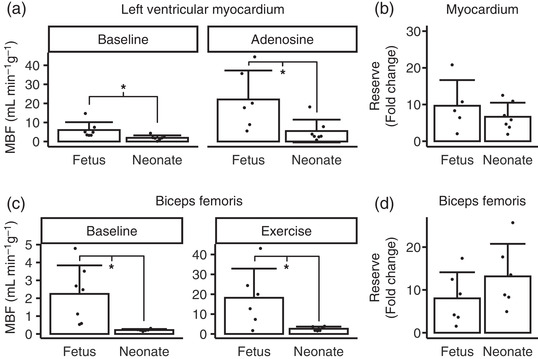
Microvascular blood flow (MBF) and reserve. Microvascular flow was assessed in fetuses at 135 dGA and in neonates at 5 dPNA. (a) In the LV myocardium, neonatal microvascular flow is significantly lower than fetal at baseline (*n* = 7 fetuses, 7 neonates; Mann–Whitney *U*‐test, *P* = 0.0041) and during adenosine hyperaemia (*n* = 6 fetuses, 7 neonates; Mann–Whitney *U*‐test, *P* = 0.0140). (b) Flow reserve in the LV myocardium is not different between fetuses and neonates (*n* = 5 fetuses, 7 neonates; Mann–Whitney *U*‐test, *P* = 0.5303). (c) In the BF, neonatal microvascular flow is significantly lower than fetal at baseline (*n* = 7 fetuses, 6 neonates; Mann–Whitney *U*‐test, *P* = 0.0012) and during electrically stimulated exercise (*n* = 6 fetuses, 6 neonates; Mann–Whitney *U*‐test, *P* = 0.0411). (d) Flow reserve is not significantly different in the BF between fetuses and neonates (*n* = 6 fetuses, 6 neonates; *t*‐test, *P* = 0.2268). Column heights correspond to group means, error bars show + SD and dots represent individual values. *P < 0.05.

Microvascular flow in the LV free wall myocardium was significantly lower in neonates compared to fetuses at baseline (Figure 4a, 2.0 (1.3) vs. 6.0 (4.1) ml g^−1^ min^−1^, *P* = 0.0041) and adenosine hyperaemia (5.5 (6.0) vs. 22.1 (15.2) ml g^−1^ min^−1^, *P* = 0.0140). Microvascular flow in the BF was substantially lower in neonates compared to fetuses at baseline (Figure 4c, 0.2 (0.1) vs. 2.2 (1.6) ml g^−1^ min^−1^, *P* = 0.0012) and during electrically stimulated exercise (2.6 (1.1) vs. 18.2 (14.7) ml g^−1^ min^−1^, *P* = 0.0411).

### 3.7 Microvascular flow reserve

Microvascular flow reserve was not significantly different between neonates and fetuses in either the LV free wall myocardium (Figure 4b, 6.7 (3.8) vs. 9.7 (7.0), *P* = 0.5303) or BF (Figure 4d, 13.2 (7.6) vs. 8.0 (6.1), *P* = 0.2268).

### 3.8 Microvascular oxygen delivery

**FIGURE 5:**
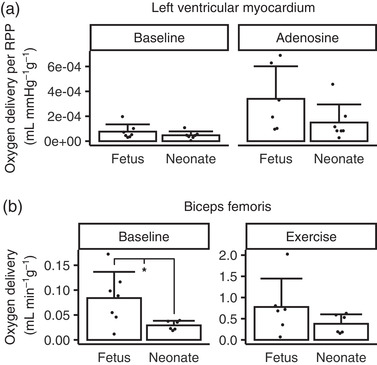
Microvascular oxygen delivery. Microvascular oxygen delivery was assessed in fetuses at 135 dGA and in neonates at 5 dPNA. (a) In the LV myocardium, oxygen delivery normalized to RPP was not significantly different at baseline (*n* = 7 fetuses, 7 neonates; *t*‐test, *P* = 0.2730) or during adenosine hyperaemia (*n* = 6 fetuses, 7 neonates; *t*‐test, *P* = 0.1545). (b) In the BF, oxygen delivery per time is lower in neonates relative to fetuses (*n* = 7 fetuses, 6 neonates; Mann–Whitney *U*‐test, *P* = 0.0350), but it is not significantly different during electrically stimulated exercise (*n* = 6 fetuses, 6 neonates; *t*‐test, *P* = 0.2175). Column heights correspond to group means, error bars show + SD and dots represent individual values. *P < 0.05.

Oxygen delivery normalized to cardiac RPP in the LV free wall myocardium was not significantly different between neonates and fetuses at baseline (Figure 5a, 4.7 × 10^−5^ (3.2 × 10^−5^) vs. 7.6 × 10^−5^ (5.9 × 10^−5^) ml mmHg^−1^ g^−1^, *P* = 0.2730) or during adenosine hyperaemia (1.5 × 10^−4^ (1.5 × 10^−4^) vs. 3.4 × 10^−4^ (2.6 × 10)^−4^ ml mmHg^−1^ g^−1^, *P* = 0.1545). Oxygen delivery rate to the BF was significantly lower in neonates compared to fetuses (Figure 5b, 0.03 (0.01) vs. 0.08 (0.05) ml min^−1^ g^−1^, *P* = 0.0350), but it was not significantly different between age groups during electrically stimulated exercise (0.38 (0.22) vs. 0.78 (0.67) ml min^−1^ g^−1^, *P* = 0.2175).

## 4 DISCUSSION

### 4.1 Perinatal changes in striated muscle microvascular physiology

Contrast enhanced ultrasound perfusion imaging describes microvascular flow in three terms: volume (MBV), flux rate (β) and their product, microvascular flow (MBF) (Kaufmann et al., 2007). MBV correlates to capillary density, while flux rate correlates to capillary diameter and inverse blood viscosity. We found that these parameters changed in striated muscles between the late‐gestation fetus and the newborn. The consequence of these changes was that, contrary to our hypothesis, there was less overall perfusion per mass after birth within both striated muscle populations studied. However, as this change was true both at rest and during hyperaemia, flow reserve was maintained between ages. Further, oxygen delivery per RPP, in the heart, was similar at rest between ages; in contrast, the oxygen delivery rate in skeletal muscle was lower in the neonate than the fetus. While numerous factors are at play during this dynamic developmental period, we postulate that the principal driver of the changes we have observed is the differential growth rates of myofibres and capillaries during late gestation and early postnatal life.

### 4.2 Microvascular blood volume and capillary growth

We found that MBV was lower postnatally than prenatally in both heart and skeletal muscle (Figure 2). Our study period spans substantial somatic and myocardial growth. While only about 17 days of developmental time separated our study groups, body weight increased by three‐quarters and heart weight more than doubled in neonates compared to fetuses (Table 1). This suggests that a major contributor to the difference between ages is changed anatomy as the growth rates of myofibres and capillaries vary.

We measured MBV both at rest and with hyperaemia. Assuming that our inductions of hyperaemia (adenosine in the myocardium, unloaded electrically stimulated exercise in the BF) recruit near‐maximum available capillary volume, our results would indicate that capillary density is decreased in neonatal relative to fetal striated muscle. While no histological study to our knowledge has been completed comparing capillary density at these developmental time points, our myocardial results do fit with broader postnatal developmental trends showing that as cardiac myofibres grow by hypertrophy, there is a gradual decline in the number of capillaries serving each fibre (Roberts & Wearn, 1941; Shipley et al., 1937). Skeletal muscle microvasculature is, in contrast, much more plastic throughout life, with angiogenesis in response to exercise occurring even in adulthood (Malek et al., 2010; McCall et al., 1996; Olfert & Birot, 2011). There is some evidence that, unlike in myocardium, the capillary to fibre ratio in skeletal muscle increases throughout childhood (Sallum et al., 2013). While those results arguably contradict ours, they did not specifically investigate the perinatal period.

### 4.3 Flux rate, haematocrit, and microvascular function

Flux rate differences with developmental stage varied between the muscle groups studied, with faster myocardial flux in the neonate than the fetus, but slower BF flux in the neonate than the fetus (Figure 3). Microvascular flux defines the rate at which blood transits through the microvascular compartment (Kaufmann et al., 2007). In recent decades, the impact of blood viscosity from haematocrit on flux through the microvasculature (where blood becomes non‐Newtonian) has become increasingly clear (Pries et al., 1994); this is underscored by our group's work on fetal anaemia demonstrating the central role that decreased viscosity from haematocrit plays in increasing flow (Davis et al., 1999; Jonker et al., 2016). Here, we saw that, as expected, neonatal haematocrit was about 40% lower than fetal (Table 2), meaning that all else being equal we should have seen increased neonatal flux rates, and indeed we did in the myocardium (Figure 3a). However, flux rates were substantially slower in neonatal relative to fetal BF (Figure 3b). That this pattern persisted during electrically stimulated exercise hyperaemia when vasodilatation has occurred implies that the explanation is not a change in basal vascular tone. Rather, this may be a manifestation of the actively maturing microvascular network within the skeletal muscle from one of short, presumably wider interconnected channels between terminal arterioles to one of complex meshes running parallel to muscle fibres (Latroche, Gitiaux et al., 2015; Smith et al., 1989; Stingl & Rhodin, 1994).

### 4.4 Microvascular perfusion and oxygen delivery

By computing the product of MBV and flux rate, we saw that microvascular flow in striated muscle was lower postnatally than prenatally in all conditions studied (Figure 4a,c). This result can be compared to earlier quantifications of myocardial perfusion in mid to late gestation and early neonatal life, performed using the radioactive microsphere technique. Dalshaug et al. (2002) compared 100–128 dGA fetal lambs and found a decrease in myocardial perfusion with advancing gestational age. In contrast to our study, Fisher et al. (1982) found perfusion of the LV free wall was significantly greater postnatally (4–23 dPNA lambs; predominantly older than ours) than prenatally (123 dGA fetuses; 12 days younger than ours). Putting our results in the context of Fisher's and Dalshaug's, we may be observing the tail end of a late gestational and perinatal decline in resting coronary perfusion which reverses itself in later postnatal life when hypertrophic growth of the LV begins to outpace that of the rest of the heart (Jonker et al., 2015). To our knowledge, other ontogenic studies of perinatal skeletal muscle perfusion have not been reported.

The most straightforward explanation for the decreased overall perfusion we identified to both striated muscle populations is the increased oxygen content of neonatal relative to fetal arterial blood (Table 2). Indeed, when we calculated oxygen delivery per RPP in the myocardium we found no significant differences between age groups (Figure 5a). In contrast, we did see lower oxygen delivery to neonatal relative to fetal skeletal muscle (Figure 5b). This may be due to a greater degree of parallel non‐nutritive perfusion of the fetal than neonatal skeletal muscle (Clark et al., 2000); measurement of local venous oxygen content enabling the calculation of oxygen consumption by the muscle could answer this question but was technically infeasible for this study. That the difference in oxygen delivery between age groups is eliminated by electrically stimulated exercise, which should increase oxygen consumption, supports this explanation.

### 4.5 Flow reserve and the limitations of anaesthesia

When using hyperaemia to explore the anatomical limits of flow, we found that maximal flow in both heart and skeletal muscle remained lower in neonates than fetuses, similar to the relationship between the ages at baseline, such that flow reserve was maintained, albeit with substantial variability (Figure 4b,d). Clinical measurements of coronary flow reserve in healthy adults range substantially, generally from 2 to 4 (Lim et al., 2000). Notably, our flow reserve values are much higher than that, possibly because of the low baseline measurements collected due to the influence of anaesthesia. The baseline fetal RPPs we measured during our studies (Table 3) are approximately 20% lower than what we have reported for age‐matched control unanaesthetized fetuses (Jonker et al., 2020), while our anaesthetized neonatal baseline RPPs are about two‐thirds lower than we have reported for week‐old control unanaesthetized neonatal lambs (Wilburn et al., 2018).

Our use of anaesthesia during CEUS studies was likely the most significant limitation of this project. We made the decision to use anaesthesia, as we have previously (Jonker et al., 2016), to enable exteriorization of fetuses for imaging, ensuring access to the LV free wall and insertion of pacing probes in the BF. We extended the use of anaesthesia to the neonatal cohort to avoid the introduction of a confounding variable between age groups.

### 4.6 Clinical relevance

This is a study of normal development meant to elucidate changes occurring during the dynamic growth period bordering parturition. For ethical reasons, comparable clinical data to these do not exist. However, these results have clinical relevance: insufficiency of the microvasculature in skeletal muscle is a driver of conditions including insulin resistance and sarcopenia, while in the myocardium its consequences include angina pectoris and cardiomyocyte death (Dorbala et al., 2014; Latroche, Matot et al., 2015; Mygind et al., 2016; Paulus & Tschöpe, 2013). Given the known impact of developmental programming on the microvasculature (Brown & Hay, 2016; Clough, 2015; Serné et al., 2000), our findings, which show decreases in microvascular perfusion without changes in flow reserve in multiple striated muscle beds in a clinically relevant large animal model, provide important context to future investigations of perturbations during this sensitive developmental period.

### 4.7 Conclusions

Microvascular perfusion of both myocardium and skeletal muscle is lower in neonatal relative to fetal lambs. Given that these differences are driven by MBV, and that this study period is one of rapid somatic and myocardial growth, we posit that our observed changes are overwhelmingly driven by faster relative growth of myofibres than their associated capillaries, such that oxygen delivery to the myocytes is maintained following initiation of air breathing.

## AUTHOR CONTRIBUTIONS

S.L., L.B., J.L. and S.J. were involved in the conception and design of this project. M.H., S.L., S.A., T.B., M.M., J.L. and S.J. were involved in data acquisition. M.H. performed data analysis and drafted the manuscript. All authors participated in critical revisions. All authors have read and approved the final version of this manuscript and agree to be accountable for all aspects of the work in ensuring that questions related to the accuracy or integrity of any part of the work are appropriately investigated and resolved. All persons designated as authors qualify for authorship, and all those who qualify for authorship are listed.

## CONFLICT OF INTEREST

T.B. is employed by Lantheus, Inc. No other authors have competing interests to declare.

## Supporting information

Statistical Summary Document

## Data Availability

Individual data are displayed in figures. As all of our sample sizes are below 30, no supplemental or repository data are provided.
